# Outcome, Complications, and Survival of Sarcomas of the Extremities Treated With Mega Prostheses: A Comprehensive Analysis of 115 Cases in a Cancer-Dedicated Hospital

**DOI:** 10.7759/cureus.71749

**Published:** 2024-10-18

**Authors:** Khalil Farooque, Muhammad bilal Shafiq, Muhammad Zoha Farooq, Ilyas Rafi, Ahmed Shoaib, Shahzaib Asif

**Affiliations:** 1 Surgical Oncology, Shaukat Khanum Memorial Cancer Hospital and Research Centre, Lahore, PAK

**Keywords:** bone sarcoma, bone tumor and limb salvage, cancer survival, joint prosthesis survivorship, surgical complication

## Abstract

Introduction: In the late 20th century, limb salvage surgery emerged as a game-changer for treating musculoskeletal tumors, evolving with advanced techniques to offer both survival and functional preservation. The objective of the study is to discuss key metrics, such as overall survival, metastasis-free survival, local recurrence-free survival, and functional outcomes, recognizing the importance of these measures. Despite hurdles such as potential infections and implant issues, it is crucial to understand and address complications, which is also highlighted in this study.

Methods: This was a retrospective review of patients with primary malignant bone tumors from January 2017 to December 2023 and the progress was followed up till June 2024. Variables assessed included patient age, size and anatomical location of the primary tumor, metastasis, histological subtype, stage and grade of tumor, resection margins, treatment provided, functional outcome, and complications. The data were analyzed with SPSS version 27. Five-year metastasis-free survival, recurrence-free survival, and overall survival were analyzed by using the Kaplan-Meier method.

Results: A total of 115 patients were analyzed, among which the most common type of tumor was osteosarcoma, found in 79 (68.7%) patients. Out of the 115 patients, 20 (17.4%) expired, six were lost to follow-up, and the five-year overall survival rate was approximately 78% (95% CI: 69.6-87.5%). Fourteen (12.2%) patients had local recurrence, and the five-year recurrence-free survival rate was approximately 83.2% (95% CI: 74.4-92.6%). Meanwhile, 27 (23.5%) patients had distant metastasis, and the five-year metastasis-free survival rate was approximately 67.5% (95% CI: 55.3-82.3%). Complications occurred in 31 (27%) patients, with 13 (11.3%) patients having local soft-tissue-related complications.

Conclusion: We observed good survival and functional outcomes in patients treated with wide-margin excision of bone sarcomas and reconstruction with mega prostheses.

## Introduction

The development of limb salvage surgery marks a key advancement in how musculoskeletal tumors are treated. This method, which emerged in the latter part of the 20th century, has undergone constant refinement. Through improved surgical methods, better prosthetic technology, and enhanced supplementary treatments, limb salvage surgery has become increasingly successful [1,2]. It has completely changed the prospects of individuals with malignant bone and soft-tissue tumors, providing them with hope for survival and preservation of functionality [3].

It is vital to measure the success of limb salvage surgeries through quantitative assessment, which involves looking at specific numbers and data to understand how well these procedures work. Different factors, such as overall survival, disease-free survival, recurrence-free survival, and functional outcomes after recovery, are crucial for determining the effectiveness of limb salvage surgery [4-6].

Although limb salvage surgery offers many benefits, it also comes with its own set of challenges, such as wound infection, mechanical and non-mechanical complications of the implant used, and difficulties in maintaining full functionality. Therefore, careful attention and quick action are needed to address any issues that may come up [7,8]. It is crucial to thoroughly study these complications to understand and mitigate the possible risks involved.

Knowledge of limb salvage surgery and possible complications that may arise in hospitals dedicated to treating cancer patients is crucial for providing individualized care. This understanding allows doctors to customize treatment plans, ensure patients fully understand what to expect, and provide a clear picture of what functional abilities patients might regain and possible complications [9]. Ultimately, this knowledge empowers patients to make informed decisions alongside their healthcare providers, leading to a better overall experience and higher satisfaction for those undergoing these intricate surgical procedures.

In light of the evolving landscape of musculoskeletal oncology, a comprehensive analysis of limb salvage surgery outcomes and complications in a cancer-dedicated hospital is a critical research endeavor. Therefore, the objective of our study was to evaluate the oncological and reconstructive functional outcomes of bone sarcoma patients treated with wide-margin excision and reconstruction with tumor prosthesis. Examining a substantial cohort of 115 cases, this study seeks to contribute valuable insights to clinical practice, enhance patient care, and further optimize the management of malignant bone tumors.

## Materials and methods

This study was a retrospective review of all patients with primary malignant bone tumors who underwent limb salvage surgery using mega prosthesis, at the Department of Surgical Oncology at Shaukat Khanum Memorial Cancer Hospital & Research Centre, Lahore, Pakistan, from January 2017 to December 2023. The study was approved by the hospital’s Institutional Review Board (approval number: EX-04-10-23-02). We carefully examined the medical records of these patients and tracked their progress until June 2024. A total of 115 patients of all age groups were included in the study. The following variables were assessed: age of the patient, size and anatomical location of the primary tumor, metastasis, recurrence, treatment provided, histological subtype, stage and grade of tumor, resection margins, complications, and outcome. All this information was meticulously recorded with a pre-designed form.

After completion of the imaging workup and histological diagnosis, the patients were discussed in the hospital’s multi-disciplinary tumor board (MDT), and treatment was devised according to the tumor stage. Patients with high-grade (grades II and III) tumors received neo-adjuvant chemotherapy, whereas sarcomas not sensitive to chemotherapy or radiotherapy were directly sent for local control (surgery). After neo-adjuvant therapy and reassessment scan, all the patients were again discussed in MDT to consider the possibility of surgical resection of the residual tumor, with the intent of obtaining clear margins. After local control, adjuvant treatment was started within three weeks if required.

**Figure 1:**
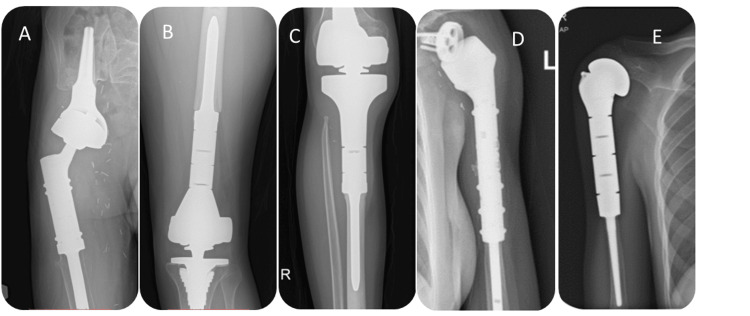
Types of mega prostheses: (A) proximal femur replacement with a LUMiC Cup, (B) distal femur replacement, (C) proximal tibia replacement, (D) proximal humerus replacement with reverse shoulder arthroplasty, and (E) proximal humerus replacement without glenoid reconstruction.

For each patient, limb salvage surgery with mega prostheses was performed to preserve limb function while effectively treating primary malignant bone tumors. The mega prostheses used in these surgeries were carefully selected based on individual patient factors, tumor characteristics, and functional requirements. Surgical techniques employed included thorough tumor resection with wide margins, followed by precise placement of the mega prosthesis to restore limb stability and function (Figure 1).

To evaluate how well patients were doing after treatment, we used the Musculoskeletal Tumor Society (MSTS) scoring system. This system characterizes different aspects of patient functionality by giving numerical ratings from 0 to 5 across six categories: pain and function, emotional acceptance (for both upper and lower extremities), support, walking and gait (for the lower extremity), and hand positioning, dexterity, and lifting ability (for the upper extremity). These ratings are added together, and the functional score is presented as a percentage of the highest possible score. Then, the results are graded based on the following scale: excellent 75-100%, good 70-74%, moderate 60-69%, fair 50-59 %, and poor ≤50% [10]. Post-surgery complications and patient status either with local disease or metastatic disease were noted.

The data were entered and analyzed in IBM SPSS Statistics for Windows, Version 27.0 (released 2020, IBM Corp., Armonk, NY). Frequency and percentages were calculated for categorical variables, and mean ± standard deviation (SD) was obtained for continuous variables. Kaplan-Meier survival was employed for overall, metastasis- and recurrence-free survival analysis. Five-year survival rates were also calculated with their respective confidence intervals. A p-value of ≤0.05 was considered statistically significant.

## Results

The study included 115 patients, of whom the majority, 74 (64.3%), were male. The average age of the patients was 22.15 ± 12.26 years (range: 12-64 years), as seen in Table 1. Regarding oncological outcome, margins were more than 1 mm in the majority of patients, 99 (85.1%), whereas close margins (<1 mm) were present in 16 (13.9%) patients.

**Table 1:** Patient demographics. DLBCL: diffuse large B-cell lymphoma

Characteristics	Category	Frequency (N)		Percentage (%)
Gender	Male		74	64.3
	Female		41	35.7
Age (years)	12–18		70	60.87
	19–50		39	33.92
	>50		6	5.21
Type of tumor	Osteosarcoma		79	68.7
	Ewing sarcoma		17	14.8
	Chondrosarcoma		14	12.2
	Others (DLBCL, plasmacytoma, spindle cell sarcoma and metastasis)		5	4.3
Site of tumor	Distal femur		57	49.6
	Proximal tibia		31	27
	Proximal femur		13	11.3
	Proximal humerus		11	9.6
	Pelvis		3	2.6

**Figure 2:**
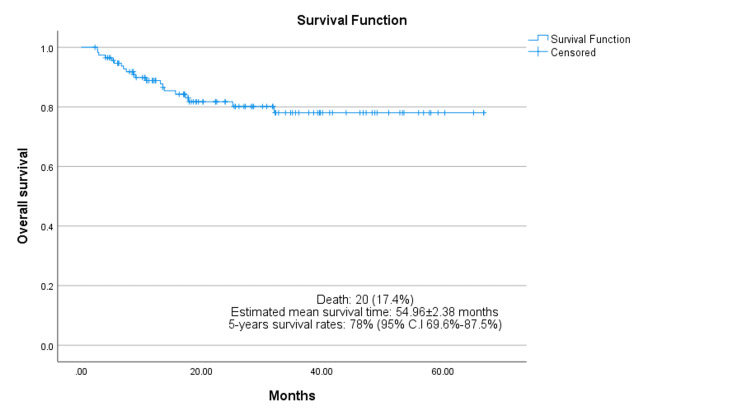
Kaplan–Meir analysis of overall survival.

Among the 115 patients examined, 20 (17.4%) expired, and six (5.2%) were lost to follow-up, but these individuals remained disease-free through their last documented follow-up. The estimated mean overall survival time was approximately 54.96 ± 2.38 months. The five-year overall survival rate was estimated to be approximately 78% (95% CI: 69.6-87.5%), as seen in Figure 2. The margin of resection exhibited no statistically significant correlation with the overall survival of patients (*p = 0.223*); however, the percentage of tumor necrosis had a statistically significant correlation (*p = 0.045*). Out of 20 patients who expired, 12 deaths were due to pulmonary metastasis, five were due to chemotherapy-related complications, and one was due to implant-related complications (bacteremia).

**Figure 3:**
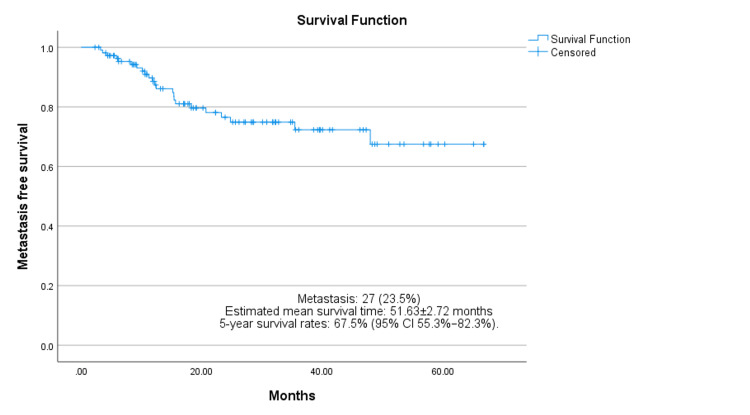
Kaplan–Meir analysis of metastasis-free survival.

Metastasis occurred in 27 (23.5%) patients. The estimated metastasis-free mean survival time was 51.63 ± 2.72 months. The five-year metastasis-free survival rate was approximately 67.5% (95% CI: 55.3-82.3%), as seen in Figure 3. We identified a statistically significant correlation between metastasis and tumor necrosis (*p = 0.017*), as well as between metastasis and resection margins (*p = 0.044*).

**Figure 4:**
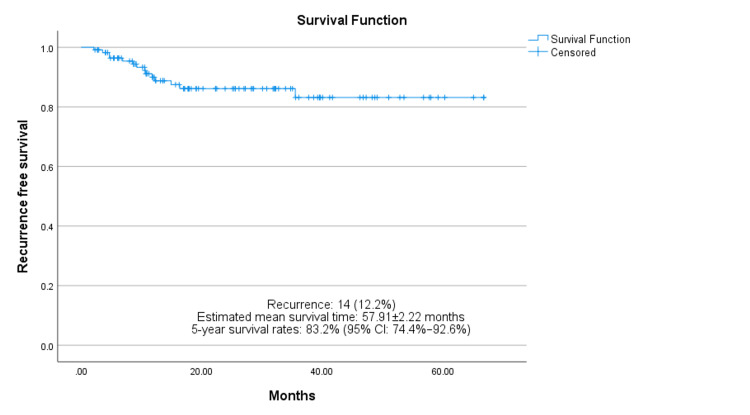
Kaplan–Meir analysis of recurrence-free survival.

Recurrence occurred in 14 (12.2%) patients. The estimated recurrence-free mean survival time was 57.91 ± 2.22 months. The five-year recurrence-free survival rate was approximately 83.2% (95% CI: 74.4-92.6%), as seen in Figure 4. Moreover, close margins exhibited a positive correlation with recurrence (*p = 0.019*).

**Figure 5:**
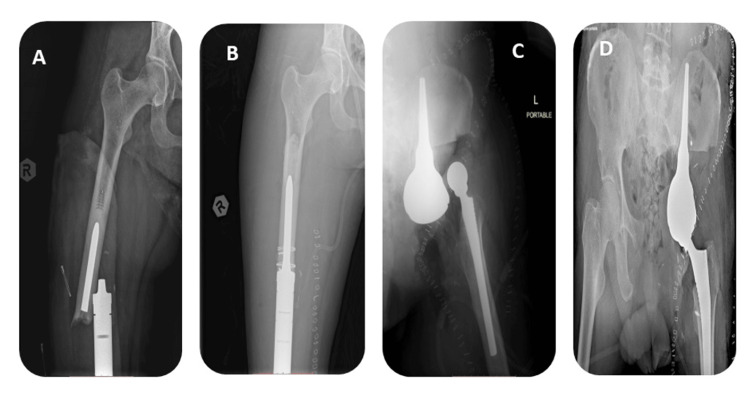
Complications of mega prostheses: (A) prosthetic fracture, (B) revision surgery, (C) dislocation of the femoral head from the LUMiC cup, and (D) post open relocation.

Complications occurred in 31 (27%) patients, with local soft-tissue-related complications in 13 (11.3%) patients, prosthetic joint infection in 11 (9.6%) patients, foot drop in three (2.6%) patients, implant dislocation in two (1.7%) patients, peri-prosthetic fracture in one (0.9%) patient, and intra-operative vascular injury in one (0.9%) patient (Figure 5). For the management of complications, two patients required open reduction (Fig.ure5C, 5D), four patients had two-stage revision surgery for prosthetic joint infection (PJI), seven patients required debridement and retention of the implant, among which two required additional flap coverage, one patient had revision surgery for fracture (Figure 5A, 5B), and two patients required amputation.

MSTS functional score was assessed in 11 patients who received upper limb mega prostheses, yielding a mean score of 23.9. Excellent to good outcome was achieved in eight (72.7%) patients. The MSTS functional score was assessed in 78 patients who received lower limb mega prostheses, yielding a mean score of 24.4. Excellent to good outcome was achieved in 47 (60.2%) patients.

## Discussion

With the advancement in diagnostic and treatment modalities for extremity sarcomas, limb salvage surgery has been the mainstay of surgical resection with curative intent. In the past few decades, multiple methods of limb reconstruction post-tumor resection have gained popularity, including reconstruction with liquid nitrogen-treated bone, irradiated allograft, vascularized bone grafts, and tumor mega prostheses [11-13]. Although each method of reconstruction has its advantages and disadvantages, mega prostheses have demonstrated better functional outcomes [14].

Improvement in chemotherapy treatment regimens has changed survival outcomes in patients with extremity bone sarcomas [15]. In our study, most patients were diagnosed with one of three high-grade sarcomas: osteosarcoma, Ewing’s sarcoma, and chondrosarcoma. In cases of osteosarcoma, all patients received neo-adjuvant methotrexate, doxorubicin, cisplatin chemotherapy as per National Comprehensive Cancer Network (NCCN) guidelines; in cases of Ewing’s sarcoma, patients received neo-adjuvant ifosfamide and etoposide as per NCCN guidelines; and in cases of chondrosarcoma, patients received upfront resection with curative intent [16-18]. Patients with tissue diagnosis of diffuse large B-cell lymphoma, plasmacytoma, spindle cell sarcoma, and metastasis received upfront local control of the disease with wide local excision and reconstruction with mega prostheses. Patients with spindle cell sarcoma and metastasis received two cycles of adjuvant radiotherapy. In cases of Ewing’s sarcoma, patients with close resection margins within 1 mm received adjuvant radiotherapy.

In cases of tumor resection and reconstruction around the knee joint, being a weight-bearing bone, reconstruction of the proximal tibia and distal femur is of paramount importance [19]. In our study, the most common sites of tumor were the proximal tibia and distal femur. When resecting proximal tibia tumors, reconstruction of the extensor mechanism is required. For proximal tibia mega prostheses, we used either a polypropylene mesh sleeve over the implant to attach the patellar tendon, which resulted in a good overall take of the tendon over the implant and preservation of knee extension, or a sleeve of bone over a hydroxyapatite-coated proximal tibia implant or integral patella clamp depending on the type of implant used. For patients receiving a proximal femur mega prosthesis, a bipolar head was used over a modular stem. No patients underwent acetabular reconstruction in cases where the tumor did not involve the acetabulum. Among the 62 patients who received lower extremities prostheses, the average MSTS score was 24.1 (80.3%), which is comparable to results published by Pala et al. (84%) and Kamal et al. (78.7%) [20,21]. The average knee range of motion was 0 to 110 degrees for distal femur mega prostheses and 0 to 90 degrees for proximal tibia mega prostheses.

Although not weight-bearing, a good, functional limb is desired when it comes to sarcomas of the upper limb [22]. In cases of wide local excision for tumors of the proximal humerus, the functional outcome mainly depends on the preservation of the rotator cuff muscles, deltoid muscle, and axillary nerve. Multiple methods have been debated in the literature for reconstruction of the resected proximal humerus, including vascularized fibula graft, allograft, and prosthetic-biological composites; however, as no method has been proven to be superior, reconstruction is tailored on an individual case basis [23]. For 11 patients with sarcomas of the proximal humerus, we used a proximal humerus mega prosthesis alone in seven patients and a reverse shoulder mega prosthesis in four patients. The average MSTS score of these patients was 23.3 (77.6%), which is comparable to results published by Denissen et al. (78%) [24]. Limitation in the abduction of the shoulder was observed in almost all patients with varying ranges of motion. Shoulder subluxation was observed in two patients with proximal humerus endoprosthesis.

In cases of proximal humerus mega prostheses, we observed no infection or wound-related complications, and smooth recovery. In cases of lower limb mega prosthesis, 13 (11.3%) patients had local soft-tissue-related complications, including skin flap necrosis and superficial infection; these complications were observed in patients who received proximal tibia mega prostheses after wide local excision of the tumor, as there is limited soft tissue coverage available for this part of the lower limb. Among these patients, two patients eventually received additional soft tissue coverage with a flap, and two patients required amputation. Eleven (9.6%) patients had PJI, of which six had distal femur mega prostheses and five had proximal tibia mega prostheses. For prosthetic joint infection, four patients were managed with serial washouts and two-stage revision of the implant, seven patients had wound debridement and serial washouts, and their implant was secured. Three (2.6%) patients had foot drop after a proximal tibia endoprosthesis, which was initially managed with an ankle foot orthosis and eventually tendon transfer after completion of oncological treatment. Two (1.7%) patients had implant dislocation, of which one patient received a proximal femur mega prosthesis and one patient received a total femur mega prosthesis. For these patients, open reduction of the dislocated implants was immediately performed, and a hip knee abduction brace was applied post reduction for three months. The cause of dislocation was extensive soft tissue dissection around the hip joint for proximal femur tumors. One (0.9%) patient had a peri-prosthetic fracture; this patient had a proximal femur mega prosthesis, and the fracture occurred in the distal femur with the stem in place. Open reduction and internal fixation with plate and cable ties were performed, and fracture healing occurred at six months. One (0.9%) patient had an intra-operative vascular injury to the femoral artery, which was intraoperatively repaired, and the limb was salvaged.

Zhang et al. reported a five-year survival rate of 71% in a similar study, which is lower than our observed rate of 78% [25]. Capanna et al. reported a five-year survival rate of 75.8%, but they excluded recurrent cases, which would further reduce the survival time [26]. In addition, a study by Umer et al. reported a survival rate of 80%, but they did not mention follow-up time in their study [27]. In our study, the most common cause of death was pulmonary metastasis. We also found a strong correlation between local recurrence and close margins (p = 0.019). Fourteen patients had local recurrence around the knee joint. Ten patients had tumor nodules away from vital structures (e.g., popliteal and femoral vessels) and were managed with wide local excision of the tissue involved. Four patients had recurrence involving vital structures, for which two patients received local radiation with second-line chemotherapy, and two patients received transfemoral amputation for local control of the disease. Rougereau et al. reported a similar treatment strategy for recurrence around the knee joint [28].

The limitations of this research article include its retrospective study design, which may introduce recall bias, and its single-center focus, which limits the generalizability of the findings to broader populations. Moreover, we have a limited variety of mega prostheses available in our country, which restricts our ability to meet patient-specific needs and achieve improved functional outcomes. A five-year follow-up provides valuable insights, but a 10-year or longer follow-up would offer a more comprehensive understanding of long-term outcomes.

We suggest developing predictive models to identify patients at higher risk of complications or failure, in order to guide personalized treatment planning. With advancing technology, exploring the role of 3D printing and robotics in improving the precision of tumor resection and prosthesis implantation can also be an interesting aspect of future research. Furthermore, we must find ways to make advanced mega prostheses and surgical techniques more accessible in low-resource settings.

## Conclusions

The mega prostheses in this study showed satisfactory results in terms of survivorship (both oncologic and reconstructive) at a minimum of five years. The multidisciplinary treatment approach for musculoskeletal tumors brings a comprehensive treatment strategy that aims to control the tumor locally and reduce the risk of metastasis, ultimately improving patient survival rates. In settings where this approach is implemented effectively, survival rates can match those observed in developed countries. However, limb salvage and reconstruction are associated with higher complication rates compared to amputation. To minimize these complications, surgeons should opt for reconstruction techniques with which they are well-versed and ensure the availability of modular options during the procedure.
